# Late‐life plasma proteins associated with prevalent and incident frailty: A proteomic analysis

**DOI:** 10.1111/acel.13975

**Published:** 2023-09-11

**Authors:** Fangyu Liu, Thomas R. Austin, Jennifer A. Schrack, Jingsha Chen, Jeremy Walston, Rasika A. Mathias, Morgan Grams, Michelle C. Odden, Anne Newman, Bruce M. Psaty, Diego Ramonfaur, Amil M. Shah, B. Gwen Windham, Josef Coresh, Keenan A. Walker

**Affiliations:** ^1^ Department of Epidemiology Johns Hopkins Bloomberg School of Public Health Baltimore Maryland USA; ^2^ Department of Epidemiology University of Washington Seattle Washington USA; ^3^ Center on Aging and Health Johns Hopkins University Baltimore Maryland USA; ^4^ Department of Medicine Johns Hopkins University Baltimore Maryland USA; ^5^ Division of Precision Medicine New York University Grossman School of Medicine New York New York USA; ^6^ Department of Epidemiology and Population Health Stanford University School of Medicine Stanford California USA; ^7^ Department of Epidemiology University of Pittsburgh Pittsburgh Pennsylvania USA; ^8^ Cardiovascular Health Research Unit, Departments of Medicine, Epidemiology, and Health Systems and Population Health University of Washington Seattle Washington USA; ^9^ Brigham and Women's Hospital, Harvard Medical School, Cardiovascular Medicine Boston Massachusetts USA; ^10^ Department of Medicine, MIND Center University of Mississippi Medical Center Jackson Mississippi USA; ^11^ Laboratory of Behavioral Neuroscience National Institute on Aging Baltimore Maryland USA

**Keywords:** aging, frailty, late life, proteomics

## Abstract

Proteomic approaches have unique advantages in the identification of biological pathways that influence physical frailty, a multifactorial geriatric syndrome predictive of adverse health outcomes in older adults. To date, proteomic studies of frailty are scarce, and few evaluated prefrailty as a separate state or examined predictors of incident frailty. Using plasma proteins measured by 4955 SOMAmers in the Atherosclerosis Risk in Community study, we identified 134 and 179 proteins cross‐sectionally associated with prefrailty and frailty, respectively, after Bonferroni correction (*p* < 1 × 10^−5^) among 3838 older adults aged ≥65 years, adjusting for demographic and physiologic factors and chronic diseases. Among them, 23 (17%) and 82 (46%) were replicated in the Cardiovascular Health Study using the same models (FDR *p* < 0.05). Notably, higher odds of prefrailty and frailty were observed with higher levels of growth differentiation factor 15 (GDF15; *p*_prefrailty_ = 1 × 10^−15^, *p*_frailty_ = 2 × 10^−19^), transgelin (TAGLN; *p*_prefrailty_ = 2 × 10^−12^, *p*_frailty_ = 6 × 10^−22^), and insulin‐like growth factor‐binding protein 2 (IGFBP2; *p*_prefrailty_ = 5 × 10^−15^, *p*_frailty_ = 1 × 10^−15^) and with a lower level of growth hormone receptor (GHR, *p*_prefrailty_ = 3 × 10^−16^, *p*_frailty_ = 2 × 10^−18^). Longitudinally, we identified 4 proteins associated with incident frailty (*p* < 1 × 10^−5^). Higher levels of triggering receptor expressed on myeloid cells 1 (TREM1), TAGLN, and heart and adipocyte fatty‐acid binding proteins predicted incident frailty. Differentially regulated proteins were enriched in pathways and upstream regulators related to lipid metabolism, angiogenesis, inflammation, and cell senescence. Our findings provide a set of plasma proteins and biological mechanisms that were dysregulated in both the prodromal and the clinical stage of frailty, offering new insights into frailty etiology and targets for intervention.

## Graphical abstract

Many plasma proteins were differentially expressed among pretrial and/or frail older adults compared to robust older adults, some of which also predicted new onset of frailty among robust/prefrail older adults. These proteins suggested metabolism, inflammation, cell senescence, and angiogenesis as important mechanisms of frailty pathogenesis.

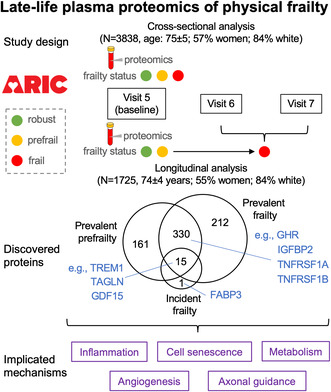

## 1 INTRODUCTION

Frailty, a syndrome of reduced reserve and increased vulnerability to stressors, predicts adverse health outcomes including mortality, hospitalization, long‐term care needs, and falls (Fried et al., 2021). In 2015, The World Health Organization (WHO) recognized frailty as an emerging public health priority (World Health Organization, 2015). Understanding the etiology of frailty can aid early recognition, prevention, and treatment of frailty, actions urged by the WHO (World Health Organization, 2015). Frailty is a complex phenotype that results from the dysregulation of multiple systems (Fried et al., 2021). Due to its multifactorial nature, attempts to identify a single mechanism are unlikely to shed light on the etiology of frailty or fully inform early intervention. This places large‐scale omics approaches that comprehensively measure molecules of a particular type (e.g., DNA, transcripts, proteins, and metabolites) in an advantageous position to study frailty as it facilitates simultaneous characterization of various processes. Moreover, agnostically exploring biological markers can more efficiently identify novel mechanisms. Plasma proteomic signatures are promising biomarkers not only because of the accessibility and routine collection of plasma in clinical settings but also because proteins, compared to genomics and transcriptomics, are closer to biological functional interpretation (Moaddel et al., 2021).

To date, four studies have assessed plasma proteomics of frailty (Table 1), three using the frailty index which defines frailty as a continuous score of cumulative deficits including physical functioning and diseases (Mitchell et al., 2023; Sathyan, Ayers, Gao, Milman, et al., 2020; Verghese et al., 2021), and the other using the physical frailty phenotype which defines prefrailty and frailty as meeting 1–2 and ≥3, respectively, of the five criteria: weight loss, weakness, slowness, exhaustion, and low physical activity (Landino et al., 2021). However, proteins associated with prefrailty need further investigation. The lack of findings in Landino et al. was likely due to the small sample size and a fairly limited measurement of the proteome. Prefrail individuals are four to five times more likely to progress to frailty than robust individuals, and are more likely to revert to a robust state than frail individuals, suggesting it is more amenable to intervention (Kojima et al., 2019). Moreover, previous longitudinal studies have focused on either the frailty index trajectory or the association between changes in proteins and frail state at the end of follow‐up (Mitchell et al., 2023; Verghese et al., 2021). To this end, proteins that predict incident frailty are not well understood.

**TABLE 1:** Current literature on proteomics of frailty.

Author (year)	Landino et al. (2021)	Sathyan, Ayers, Gao, Milman, et al. (2020))	Verghese et al. (2021)	Mitchell et al. (2023)
Sample size	752 (baseline: 302 prefrail, 45 frail; follow‐up: 52 prefrail/frail)	880	671 (stable: 220; mild frail: 260; moderate frail: 156; several frail: 35)	980 (baseline); 686 (5‐years); 318 (10 years)
Baseline mean age (years)	74	75	75	75
Study design	Cross‐sectional/longitudinal	Cross‐sectional	Longitudinal	Cross‐sectional/longitudinal
Frailty definition	Physical Frailty Phenotype	Frailty Index (41 items)	Frailty Index (41 items)	Frailty Index (13 items)
Protein platform	1.3k SomaScan	4.0k SomaScan	4.0k SomaScan	Olink Proseek Multiplex Cardiovascular II (CVD II) panel
# Proteins measured	1301	4265	4265	92
# Proteins identified	0 (cross‐sectional with prefrailty) 4 (cross‐sectional with frailty); 2 (longitudinal with time to prefrailty or frailty)	143	11	8 core proteins for cross‐sectional and longitudinal associations
*p*‐Value threshold	FDR *p* < 0.05	Bonferroni (*p* < 1.17 × 10^−5^)	Bonferroni (*p* < 1.17 × 10^−5^)	FDR *p* < 0.05
Top proteins	*Cross‐sectional* ↑ CXCL13, THBS2, ↓ CKM, CKB/CKM *Longitudinal* ↑ CDK5/CDK5R1, IL1α	↑ FABP, FABPA, leptin, ↓ ANTR2, NELL1, ERBB1	↑ FABP, FABPA, leptin, ↓ NCAN, CACNA2D3, DNER, ERBB1, ANTR2, OMGP, contactin‐1, Glypican‐3	↑ CD4, FGF23, Gal‐9, PAR‐1, REN, TNFRSF10A, TNFRSF11A, TNFRSF10B

*Note*: ↑ increased expression; ↓ decreased expression.

Abbreviations: ANTR2, anthrax toxin receptor 2; CACNA2D3, voltage‐dependent calcium channel subunit alpha‐2/delta‐3; CD4, T‐cell surface glycoprotein CD4; CDK5/CDK5R1, cyclin‐dependent kinase 5/cyclin‐dependent kinase 5 regulatory subunit 1; CKB, creatine kinase B; CKM, creatine kinase type M; CXCL13, chemokine (C‐X‐C motif) ligand 13; DNER, delta and Notch‐like epidermal growth factor‐related receptor; ERBB1, epidermal growth factor receptor; FABP, fatty acid‐binding protein, heart; FABPA, fatty acid‐binding protein, adipocyte; FGF23, fibroblast growth factor 23; Gal‐9, Galectin‐9; IL1α, interleukin 1 alpha; NCAN, neurocan core protein; NELL1, neural EGF Like‐Like molecule 1; OMGP, oligodendrocyte‐myelin glycoprotein; REN, renin; THBS2, thrombospondin‐2; TNFRSF, tumor necrosis factor receptor superfamily.

In this study, we used a version of the SomaScan platform that quantifies nearly 5000 proteins on 3838 older adults aged ≥65 years in the Atherosclerosis Risk in Community (ARIC) study) to identify plasma proteins that are (1) associated with both physical prefrail and frail status and (2) predict incident frailty among robust or prefrail older adults. The larger sample size and the broader proteomic measurement provide greater power and coverage to better capture the proteins that are potentially relevant to frailty risk. We validated prefrailty‐ and frailty‐associated proteins in an independent sample (the Cardiovascular Health Study [CHS]).

## 2 METHODS

### 2.1 Discovery cohort

**FIGURE 1:**
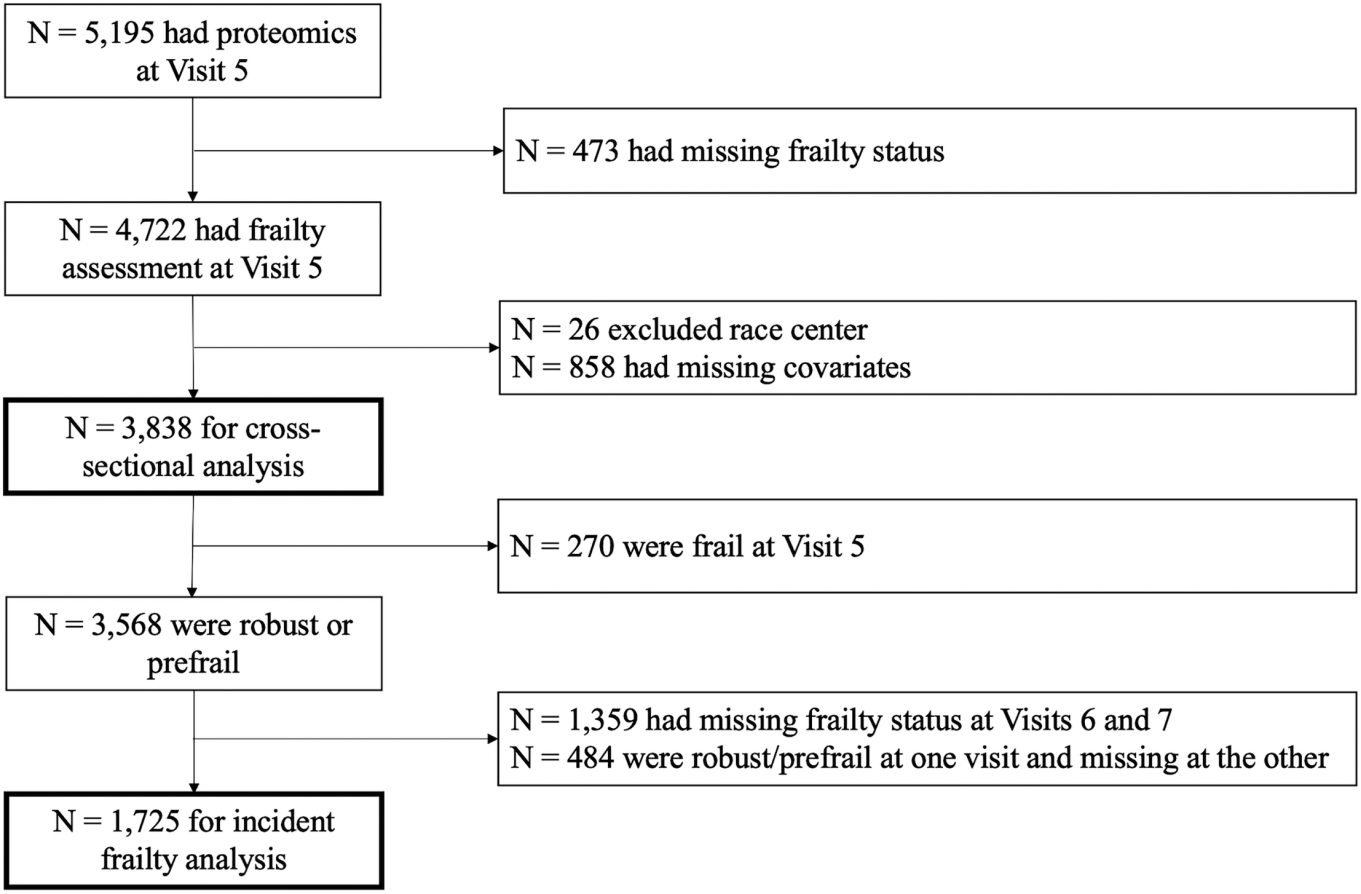
Flowchart of ARIC participants selection.

The ARIC study is an ongoing community‐based cohort study originally designed to understand the etiology of atherosclerosis and its clinical consequences during midlife (Wright et al., 2021), but as the cohort aged, it provides unique opportunities to investigate risk factors in mid‐life to aging trajectories. The participants were enrolled from four communities across the United States: Washington County, MD; Forsyth County, NC; northwestern suburbs of Minneapolis, MN; and Jackson, MS. In this analysis, we used data from visits that included frailty assessments: Visit 5 (in 2011–2013), Visit 6 (in 2016–2017), and Visit 7 (in 2018–2019), with Visit 5 being the baseline and Visits 6 and 7 being the follow‐up visits. Proteomics were assessed at baseline. The analytical sample for the cross‐sectional analysis consisted of 3838 participants who had proteomics and frailty assessments and complete covariates at baseline (Figure 1). Our sample excluded self‐identified non‐Black and non‐White participants or self‐identified Black participants at the Washington County and Minneapolis study sites due to small sample sizes (*n* = 26). The sample for the longitudinal analysis consisted of 1725 participants after excluding participants who were frail at baseline (*n* = 270), had missing frailty status at both follow‐up visits (*n* = 1359), and was robust or prefrail at one follow‐up visit and missing at the other follow‐up visit (*n* = 484, Figure 1).

### 2.2 Replication cohort

The CHS is a population‐based cohort study of cardiovascular disease in community‐dwelling older adults ≥65 years from 4 communities: Sacramento County, CA; Washington County, MD; Forsyth County, NC; and Allegheny County, PA (Fried et al., 1991). Proteomics and frailty status were measured for participants from the 1992 to 1993 visit (Y5, baseline). Frailty status at follow‐up was assessed at the 1996–1997 visit (Y9). We had 2570 participants for the replication of cross‐sectional results, and 1817 participants for replication of longitudinal results.

### 2.3 Proteomics measurement

Relative abundances of the plasma proteins and protein complexes were measured by the SomaScan platform (Version 4.0; Somalogic, Inc.). The SomaScan platform uses single strands of DNA with chemically modified nucleotides, called modified aptamers or “SOMAmers”, which act as protein‐binding reagents with defined three‐dimensional structures and unique nucleotide sequences. The abundances of the SOMAmers were quantified using dynamic DNA detection technology and represented the levels of the proteins in plasma. The assay was shown to have a sensitivity comparable to the conventional immunoassay approaches and good reproducibility (Walker et al., 2021). All ARIC participants were measured using Version 4.0 of the SomaScan in ARIC. A total of 4995 SOMAmers of 4712 unique proteins passed the quality control and were used in the present study. Most of the CHS participants were measured using the same version (*n* = 2350, 91%) whereas the others were measured using Version 4.1 (assayed 7596 aptamers including all those measured by Version 4.0). The relative abundances for the SOMAmers from Version 4.1 were scaled using scaling factors provided by SomaLogic to allow for harmonization of SOMAmer measurements across the Version 4.0 and 4.1 platforms.

### 2.4 Frailty assessment

Frailty was operationalized in the ARIC study and the CHS using the five criteria of the physical frailty phenotype (Fried et al., 2001; Kucharska‐Newton et al., 2017). Weakness, slowness, exhaustion, and low physical activity were similarly defined in both cohorts. Weakness was defined as grip strength below the cut points established in the CHS. Slowness was defined as the usual gait speed below the CHS cut‐points. Exhaustion was defined as responding “some of the time” or “most of the time” to either of the two questions from the CES‐D scale: I felt everything I did was an effort, or I could not get “going.” Low physical activity was ascertained as ranking in the lowest quintile of self‐reported physical activity. In ARIC, weight loss at baseline was defined as a >10% decrease in measured weight from Visit 4, when participants were last examined in‐person and many were still middle‐aged, or had a current BMI < 18.5 kg/m^2^. At the follow‐up visits, participants were all over the age of 65 years, and the criteria of >5% lower weight from the previous visit or current BMI <18.5 kg/m^2^ was used. In the CHS, weight loss was defined as self‐report of unintentional weight loss >10 lbs (i.e., not due to dieting or exercise) at the baseline, and as ≥5% of measured unintentional weight loss at the follow‐up.

At all visits, the presence of no criteria was defined as robust; 1–2 criteria as prefrail; and 3–5 criteria as frail. ARIC participants with missing values for one criterion and meeting 0 or 2 of the non‐missing criteria and participants with missing values for more than one criterion and meeting <3 of the non‐missing criteria was assigned a missing value for the frailty status. CHS participants with >2 missing criteria were classified as missing frailty status. ARIC participants who were robust or prefrail at baseline but frail at either follow‐up visit were classified as having incident frailty. Participants who were robust or prefrail at baseline and both follow‐up visits were classified as having no incident frailty. In CHS, participants who were robust or prefrail at Y5 and frail at Y9 were in the incident frailty group, and those who were robust or prefrail at Y5 and Y9 were in the no incident frailty group.

### 2.5 Statistical analysis

We used the same models for the discovery and replication analyses. To examine the cross‐sectional associations between proteins and frailty status, we used multinomial logistic regression models with the three categories of frailty assessment as the dependent variable and the relative abundances of the SOMAmers as the main independent variable. The relative abundances were first log 2‐transformed and then further standardized to mean zero and standard deviation of one. Each multinomial logistic model produced two sets of coefficients: one estimated the odds ratio (OR) of being frail to being robust with 1 standard deviation (SD) higher abundance of the SOMAmer, and the other estimated the OR of being prefrail to being robust with 1 SD higher abundance of the SOMAmer. To test the associations between proteins and incident frailty, logistic models of incident frailty were used because frailty assessments were performed at a limited number of discrete study visits. Participants with no incident frailty were used as the reference group. For the discovery analyses, SOMAmers were considered significant at the Bonferroni level, the most rigorous and conservative threshold, if *p*‐value <1 × 10^−5^ while a false discovery rate (FDR) level with Benjamini‐Hochberg FDR adjusted *p*‐value <0.05 was used for pathway analysis where retaining greater power is important. We tested 214 SOMAmers associated with either prevalent prefrailty or prevalent frailty and 4 SOMAmers associated with incident frailty at Bonferroni level for replication. We reported proteins replicated with Benjamini‐Hochberg FDR adjusted *p*‐value <0.05.

In the discovery analyses, we progressively adjusted for covariates as follows: Model 1 (adjusted for self‐reported age, sex, race or race‐center, education, family income), Model 2 (additionally adjusted for drinking and smoking status, total cholesterol, estimated glomerular filtration rate [eGFR], history of hypertension, diabetes, coronary heart disease, heart failure, cancer, and chronic lung disease); and Model 3 (additionally adjusted for body mass index [BMI]). All covariates were measured at baseline. The detailed definitions of the covariates are summarized in Table S12. The adjustment of chronic diseases was to control for the confounding effect as chronic diseases can increase the risk of frailty (Fried et al., 2004) and may alter plasma protein level. We focused on Model 3 in this paper, but results from the other models are reported in Tables S15–S18 and the comparison between models is presented in Figures S1–S3. The replication analyses included Model 3 only. History of stroke was excluded because stroke is an exclusion criterion for frailty assessment in CHS. The replication models also included a variable that indicates the SOMAscan versions (4.0/4.1).

### 2.6 Ingenuity pathway analyses

To better interpret the biological and functional pathways represented by the discovered proteins, we performed canonical pathway analysis and upstream regulator analysis separately for proteins discovered in cross‐sectional and longitudinal analyses, using QIAGEN's Ingenuity Pathway Analysis (IPA; QIAGEN Inc). Ingenuity Knowledge Base was used as the reference set and included direct and indirect experimentally confirmed relationships from human. For cross‐sectional analysis, 490 unique proteins associated with prefrailty and 540 unique proteins associated with frailty at the FDR level were mapped to the IPA database and included in the analyses. For longitudinal analysis, 126 proteins with nominal *p*‐value <0.01 were mapped and included. A less stringent threshold for longitudinal analysis was chosen to achieve a sufficient number of proteins recommended by QIAGEN. The methods used in canonical pathway analysis and upstream regulator analysis were described previously (Walker et al., 2021). Briefly, the IPA uses a right‐tailed Fisher's exact test to quantify the probability of overlap due to random chance between the included proteins from this study and a set of proteins known to exist within a specific pathway or being regulated by an upstream regulator. We reported pathways and upstream regulators that have *p*‐values for enrichment <0.05 after the Benjamini‐Hochberg FDR adjustment.

## 3 RESULTS

### 3.1 Participant characteristics in the ARIC study

The baseline characteristics of the participants included in the cross‐sectional and longitudinal analyses are summarized in Table 2 and Table S13. Of the 3838 participants included in the cross‐sectional analysis, 270 participants (7.0%) were frail and 1806 participants (47.1%) were prefrail. Compared to robust participants, prefrail and frail participants were older, more likely to be women or self‐report race as Black, had lower education level and family income, had lower total cholesterol, lower eGFR, higher BMI, and were more likely to have chronic diseases.

**TABLE 2:** The ARIC participant characteristics at baseline.

	Cross‐sectional sample (*N* = 3838)			Longitudinal sample (*N* = 1725)	
	Robust	Prefrail	Frail	No incident frailty	Incident frailty
Mean (SD)/*N* (%)	*n* = 1762	*n* = 1806	*n* = 270	*n* = 1484	*n* = 241
Age, years	74.3 (4.5)	76.3 (5.2)	77.8 (5.7)	73.8 (4.3)	75.8 (4.9)
Women	948 (53.8%)	1050 (58.1%)	181 (67.0%)	817 (55.1%)	140 (58.1%)
Race center					
Minneapolis Whites	661 (37.5%)	551 (30.5%)	61 (22.6%)	496 (33.4%)	69 (28.6%)
Jackson Blacks	219 (12.4%)	280 (15.5%)	53 (19.6%)	204 (13.7%)	43 (17.8%)
Washington Whites	508 (28.8%)	545 (30.2%)	92 (34.1%)	413 (27.8%)	85 (35.3%)
Forsyth Blacks	15 (0.9%)	26 (1.4%)	6 (2.2%)	22 (1.5%)	2 (0.8%)
Forsyth Whites	359 (20.4%)	404 (22.4%)	58 (21.5%)	349 (23.5%)	42 (17.4%)
Total cholesterol, mmol/L	4.7 (1.1)	4.6 (1.1)	4.5 (1.0)	4.7 (1.0)	4.5 (1.0)
eGFR, mL/min/1.73 m^2^	68.9 (16.4)	64.1 (17.8)	54.0 (19.0)	70.6 (15.7)	60.0 (17.6)
Hypertension	1222 (69.4%)	1365 (75.6%)	222 (82.2%)	999 (67.3%)	197 (81.7%)
Diabetes	444 (25.2%)	634 (35.1%)	127 (47.0%)	356 (24.0%)	112 (46.5%)
Coronary heart disease	201 (11.4%)	307 (17.0%)	58 (21.5%)	146 (9.8%)	42 (17.4%)
Heart failure	10 (0.6%)	28 (1.6%)	14 (5.2%)	8 (0.5%)	4 (1.7%)
Stroke	36 (2.0%)	64 (3.5%)	23 (8.5%)	29 (2.0%)	9 (3.7%)
Cancer	369 (20.9%)	430 (23.8%)	74 (27.4%)	279 (18.8%)	57 (23.7%)
Lung disease	329 (18.7%)	449 (24.9%)	99 (36.7%)	269 (18.1%)	74 (30.7%)
BMI, kg/m^2^	28.2 (5.0)	28.7 (5.6)	30.1 (7.1)	28.2 (4.9)	31.1 (6.3)

Abbreviations: BMI, body mass index; eGFR, estimated glomerular filtration rate.

Among the 1725 participants included in the longitudinal analysis, 241 participants (14.0%) had incident frailty. Compared to participants who did not develop frailty, participants who had incident frailty were also older and had lower total cholesterol, lower eGFR, higher BMI, and more chronic diseases at baseline (Table 1).

### 3.2 Proteome‐wide analysis of prevalent prefrailty and frailty in the ARIC study

**FIGURE 2:**
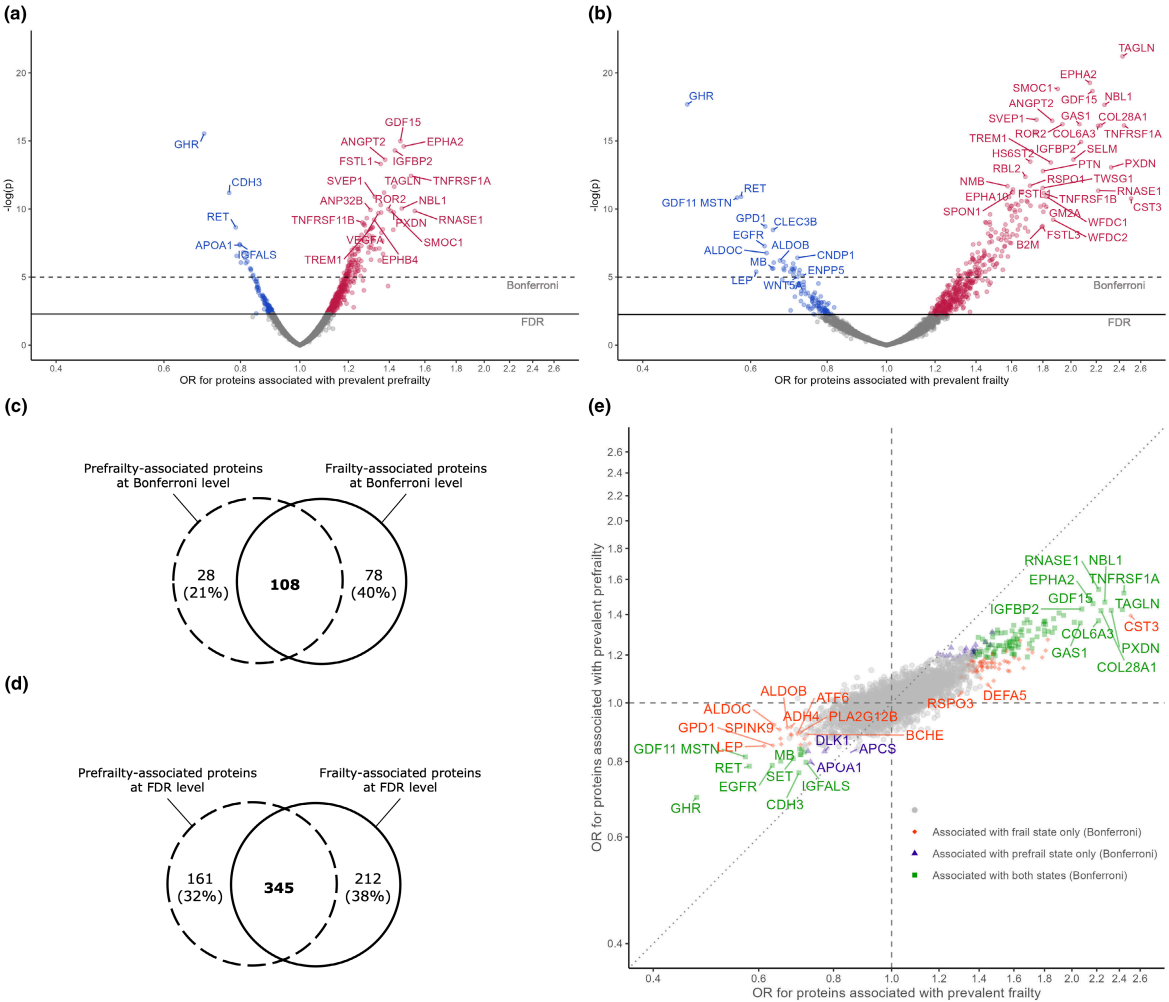
Proteins associated with baseline prefrailty (a) and frailty (b) in ARIC, the number of proteins associated with both states at Bonferroni level (c) and at FDR level (d), and consistency of associations with two frailty states (e) from the multinomial logistic regression models adjusted for age, sex, race‐center, education, family income, drinking status, smoking status, body mass index, total cholesterol, estimated glomerular filtration rate, and history of hypertension, diabetes, coronary heart disease, heart failure, stroke, cancer, and lung disease. Top proteins were annotated with entrez gene symbol.

A total of 136 and 186 SOMAmers (134 and 179 unique proteins) were cross‐sectionally associated with prefrailty and frailty, respectively, after the Bonferroni correction (*p* < 1 × 10^−5^). A total of 506 and 557 SOMAmers (495 and 544 unique proteins) were associated with prefrailty and frailty, respectively, at the FDR level (Figure 2, Tables S1 and S2). The top five proteins associated with higher odds of prefrailty included growth differentiation factor 15 (GDF15; OR per 1 SD increase = 1.46), ephrin type‐A receptor 2 (EPHA2; OR = 1.48), insulin‐like growth factor‐binding protein 2 (IGFBP2; OR = 1.43), angiopoietin‐2 (ANGPT2; OR = 1.38), and follistatin‐related protein 1 (FSTL1; OR = 1.36). The top 5 proteins associated with lower odds of prefrailty included growth hormone receptor (GHR; OR = 0.70), Cadherin‐3 (CHD3; OR = 0.77), proto‐oncogene tyrosine‐protein kinase receptor Ret (RET; OR = 0.79), insulin‐like growth factor‐binding protein complex acid labile subunit (IGFALS; OR = 0.80), and apolipoprotein A‐1 (APOA1; OR = 0.80).

Prefrailty‐associated proteins GDF15 (OR = 2.17), EPHA2 (OR = 2.15), GHR (OR = 0.47), and RET (OR = 0.58) were also among the top proteins associated with frailty. Other top proteins associated with higher odds of frailty included transgelin (TAGLN; OR = 2.43), SPARC‐related modular calcium‐binding protein 1 (SMOC1; OR = 1.90), and neuroblastoma suppressor of tumorigenicity 1 (NBL1; OR = 2.27). Growth differentiation factor 11/8 (GDF11 MSTN; OR = 0.57), glycerol‐3‐phosphate dehydrogenase [NAD+], cytoplasmic (GPD1; OR = 0.63), and tetranectin (CLEC3B; OR = 0.65) are among other top proteins associated with lower odds of frailty.

At the Bonferroni level, 108 SOMAmers (106 unique proteins) were associated with both prefrailty and frailty (79% and 58% of the prefrailty‐ and frailty‐associated SOMAmers, respectively, Figure 2c). At the FDR level, 345 SOMAmers (337 unique proteins) were associated with both states (68% and 62% of the prefrailty‐ and frailty‐associated proteins, respectively, Figure 2d). The overlap of prefrail‐ and frailty‐associated SOMAmers was greater among those associated with higher odds of both states compared to SOMAmers associated with lower odds (Figure S4, Fisher's exact test *p* < 0.001 for proteins passed FDR level). The associations of top proteins with prefrailty and frailty were also highly consistent (Spearman correlation = 0.75, Figure 2e).

### 3.3 Proteome‐wide analysis of incident frailty in the ARIC study

**FIGURE 3:**
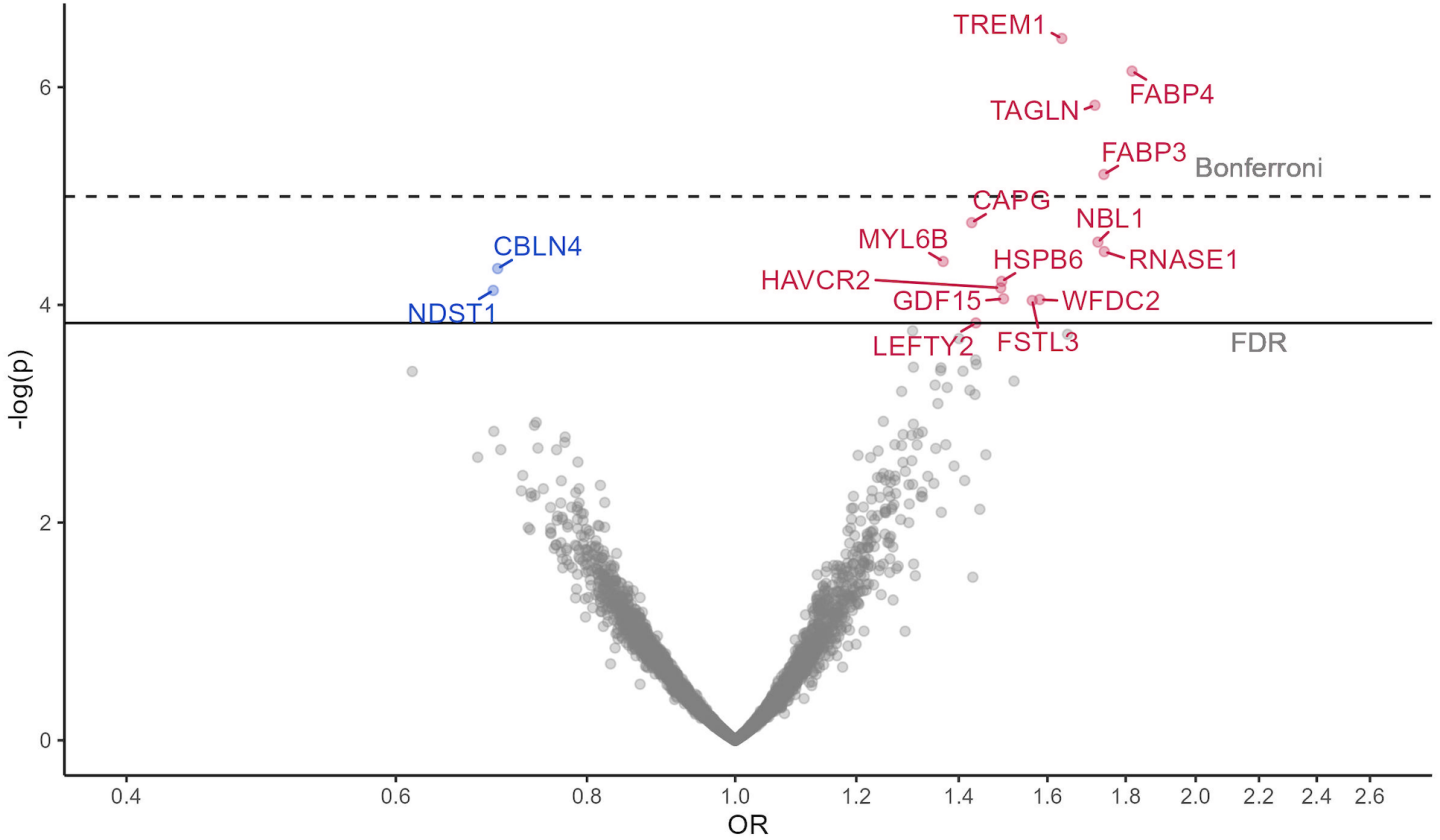
Proteins associated with incident frailty in ARIC from the logistic models adjusted for age, sex, race‐center, education, family income, drinking status, smoking status, body mass index, total cholesterol, estimated glomerular filtration rate, and history of hypertension, diabetes, coronary heart disease, heart failure, stroke, cancer, and lung disease. Top proteins were annotated with entrez gene symbol.

We found 14 SOMAmers (14 proteins) associated with higher odds of incident frailty and 2 SOMAmers (2 proteins) with lower odds of incident frailty at the FDR level (Figure 3, Table S3). Among them, triggering receptor expressed on myeloid cells 1 (TREM1, OR = 1.64), TAGLN (OR = 1.72), fatty acid‐binding protein adipocyte (FABP4, OR = 1.82), and fatty acid‐binding protein heart (FABP3, OR = 1.74) were associated with higher incident frailty at Bonferroni level (Figure 3). Except for FABP3, all discovered proteins associated with incident frailty were also cross‐sectionally associated with prefrailty or frailty at least at the FDR level (Tables S1 and S2).

### 3.4 Replication of discovered proteins in the CHS

The baseline participant characteristics in the CHS are summarized in Table S14. Of the 2570 participants included in the cross‐sectional analysis, 183 participants (7.1%) were frail, and 1269 participants (49.4%) were prefrail. Of the 1817 participants included in the longitudinal analysis, 127 participants (7.0%) had incident frailty. The participants in the CHS had a similar age distribution as the participants in the ARIC study. However, the mean BMI and the prevalence of hypertension, diabetes, cancer, and lung disease were lower in the CHS than in the ARIC study.

**FIGURE 4:**
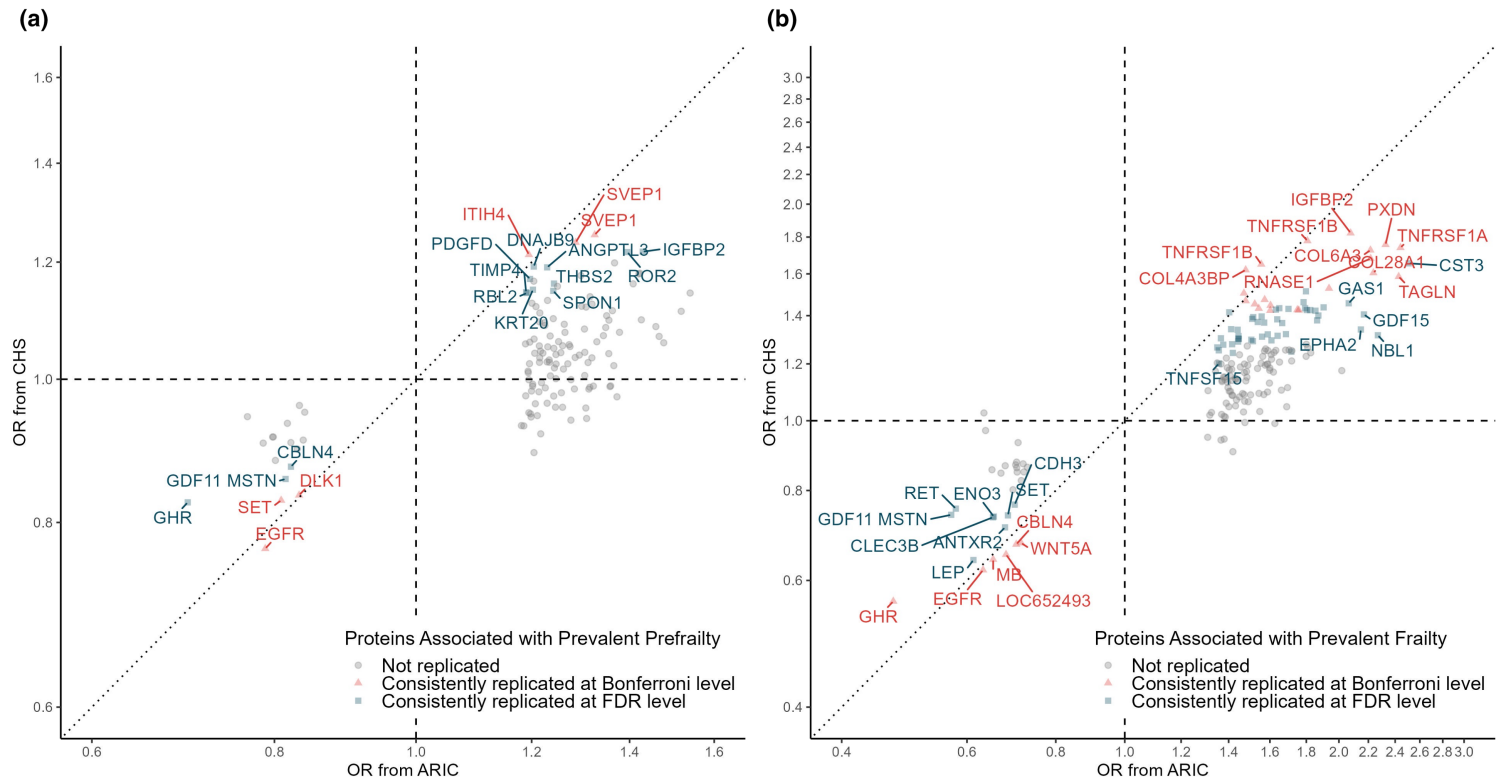
Replication in the CHS of the prevalent prefrailty proteins (a) and the prevalent frailty proteins (b). Top proteins were annotated with entrez gene symbol. Red triangle represents proteins replicated at Bonferroni level. Blue square represents proteins replicated at the FDR level. Gray circle represents proteins that are not replicated. The dashed lines represent OR = 1 in each cohort. The dotted line represents the line of agreement.

We tested the 136 SOMAmers associated with prevalent prefrailty and 186 SOMAmers associated with prevalent frailty (214 unique SOMAmers) and 4 SOMAmers for incident frailty in the CHS. A total of 24 (18%) SOMAmers (23 unique proteins) associated with prevalent prefrailty were replicated at the FDR level (Figure 4a, Table S4). A total of 84 (54%) SOMAmers (82 unique proteins) associated with prevalent frailty were replicated at the FDR level (Figure 4b). All replicated proteins had consistent directions of associations as in ARIC. The top proteins discovered in ARIC, including GHR, IGFBP2, TAGLN, and GDF15 were replicated with prefrailty and/or frailty. None of the four proteins associated with incident frailty were replicated in the CHS (Table S5), even though they had the same directions of associations.

### 3.5 Frailty‐associated proteins and chronological age

Given that a sizable proportion of the proteins we discovered, for example, TREM1, TAGLN, and GDF15, have been shown to associated with chronological age (Sathyan, Ayers, Gao, Weiss, et al., 2020), we performed a post‐hoc cross‐sectional analysis of the association between chronological age and each of the 719 proteins linked to prevalent prefrailty, prevalent frailty, and/or incident frailty at an FDR‐corrected significance level. We found 380 (53%) proteins were associated with chronological age after FDR correction, adjusting for all covariates in Model 3 (excluding frailty status; Figure S5). These findings suggest that a large proportion of the frailty‐associated proteome is represented by proteins that covary strongly with age, supporting the hypothesis that frailty risk is increased among those with advanced *biological* age.

### 3.6 Pathways and upstream regulators of the discovered proteins

**FIGURE 5:**
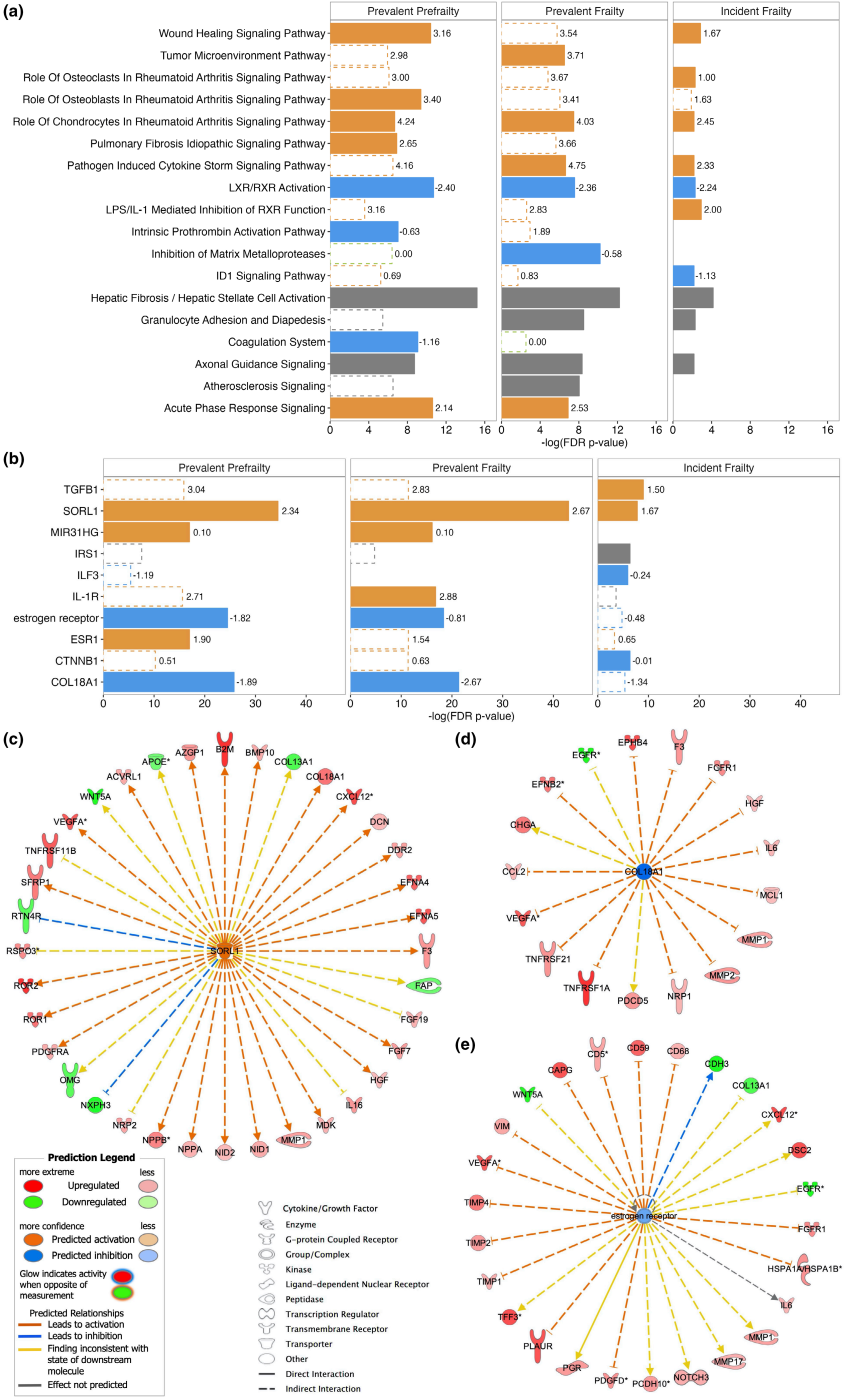
The top 10 enriched pathway (a) and top 5 upstream regulators (b) among proteins associated with prevalent prefrailty, prevalent frailty, and incident frailty, and the target proteins of SORL1 (c), COL18A1 (d), and estrogen receptor (e) in ARIC. Solid bars denote the top 10 pathways or top 5 regulators for each analysis. Open bars with a dashed outline denote pathways or regulators that are significantly enriched after FDR correction but are not among the top 10 pathways or top 5 regulators for the specific analysis. The length of the bar denotes the ‐log(*p*‐values after FDR correction). Orange color denotes pathways or regulators that are predicted to be activated (*z*‐score labeled at the end of the bar > 0). Blue color denotes pathways or regulators that are predicted to be inhibited (*z*‐score < 0). Green color denotes pathways or regulators that are predicted to be neither activated nor inhibited (*z*‐score = 0). Gray color denotes pathways or regulators whose activation/inhibition states are not predicted. TGB1, transforming growth factor beta‐1; SORL1, sortilin‐related receptor 1; MIR31HG, MIR31 host gene; IRS1, insulin receptor substrate 1; ILF3, interleukin enhancer binding factor 3; IL‐1R, interleukin‐1 receptor; ESR1, estrogen receptor 1; CTNNB1, Catenin Beta 1; COL18A1, collagen type XVIII alpha 1 chain.

The top 10 enriched pathways for the proteins associated with prevalent prefrailty, prevalent frailty, or incident frailty are shown in Figure 5a. The liver X receptors/retinoid X receptors (LXR/RXR) activation involved in cholesterol and glucose metabolism was a top inhibited pathway enriched for prevalent prefrailty, prevalent frailty, and incident frailty. Notably, pathways related to inflammation and immune activation, for example, rheumatoid arthritis signaling, pathogen‐induced cytokine storm signaling, and wound healing were activated among participants with prevalent prefrailty/frailty and incident frailty. Axonal guidance signaling, a process important for neuronal development and regeneration, was another top enriched pathway among all three analyses, though its activation/inhibition status could not be determined. The complete lists of significantly enriched pathways (FDR *p*‐value for enrichment <0.05) are summarized in Tables S6–S8. We also used our SomaScan data as the reference set in the pathway analyses. The top 10 pathways generally remained to be the top 10 pathways with smaller enrichment *p*‐values (data not shown).

The top 5 upstream regulators for the proteins associated with prevalent prefrailty, prevalent frailty, or incident frailty are shown in Figure 5b. Sortilin‐related receptor 1 (SORL1) related to lipid and beta‐amyloid metabolism was the topmost upstream regulator for proteins associated with prevalent prefrailty, prevalent frailty, and incident frailty. Collagen type XVIII alpha 1 chain (COL18A1) and estrogen receptor, regulators of proteins involved in angiogenesis and senescence‐associated secretory phenotype (SASP) factors, were among the top 5 upstream regulators for prevalent prefrailty and prevalent frailty and were significantly enriched in proteins associated with incident frailty, albeit not the top‐ranked regulators. The target proteins of SORL1, COL18A1, and estrogen receptor associated with frailty (FDR *p* < 0.05) in our sample were presented in Figure 5c–e. The full lists of significant upstream regulators (FDR *p*‐value for enrichment <0.05) and their target proteins can be found in Tables S9–S11. The top upstream regulators were also robust to the change of reference set to SomaScan data (data not shown).

## 4 DISCUSSION

We used a proteome‐wide approach to evaluate the plasma proteins associated with prevalent prefrailty, prevalent frailty, and incident frailty in a large community‐based study of older adults. After extensive adjustment and replication, we identified a large set of novel proteins that are differentially abundant in frail and prefrail individuals and four proteins associated with a higher risk of incident frailty among robust or prefrail older adults over a 6‐year follow‐up period. Pathway and upstream regulator analyses implicated several biological systems, including lipid and glucose metabolism, angiogenesis, inflammation, and cell senescence, providing new insights into the etiology of frailty and a foundation for potential targets for intervention.

Our results also provide a robust independent replication for a large proportion of the previously identified frailty biomarkers. Using comparable adjustment (Model 1), we found 100 of the 143 proteins associated with frailty index in Sathyan et al. were associated with prevalent prefrailty and/or frailty defined by physical frailty phenotype in our sample (Sathyan, Ayers, Gao, Milman, et al., 2020). We also found that all 11 proteins associated with frailty index trajectory category in Verghese et al. (2021) and 2 of the 8 core proteins longitudinally associated with frail status measured by frailty index in Mitchell et al. (2023) were associated with incident frailty in our study. Our Model 3 also replicated three of the four proteins associated with prevalent frailty in Landino et al. (2021).

Our findings also considerably expand the list of candidate proteins that predict the development of frailty by following robust and prefrail participants to identify TREM1, TAGLN, FABP3, and FABP4 as being associated with incident (new‐onset) frailty. TREM1 is a myeloid receptor involved in innate and adaptive immunity, which showed a suggestive cross‐sectional. Association (*p* = 9.82 × 10^−5^) with frailty index previously (Sathyan, Ayers, Gao, Milman, et al., 2020). Activation of this cell surface receptor by bacterial or fungal products promotes the production of inflammatory chemokines and cytokines by neutrophils and monocytes (Tessarz & Cerwenka, 2008). TREM1 has also been shown to be associated with obesity, diabetes (Subramanian et al., 2017), cardiovascular disease (Kouassi et al., 2018), cancer (Raggi & Bosco, 2020), and incident dementia (Walker et al., 2021). While it is possible that the pro‐inflammatory response triggered by higher levels of TREM1 may impact frailty risk, secreted levels of this myeloid receptor in plasma may simply be a sensitive indicator of a pro‐inflammatory milieu that has been consistently linked to frailty risk (Wang et al., 2019).

TAGLN is an actin‐binding/gelling protein also associated with incident frailty, is mainly expressed in smooth muscle cells and involved in smooth muscle cell differentiation, calcium‐independent contraction, and cell motility through the regulation of cytoskeletal organization (Elsafadi et al., 2020). TAGLN was also found to be elevated in senescent cells (Basisty et al., 2020). However, TAGLN and its isoform, TAGLN‐2, have not been previously associated with physical frailty phenotype or frailty index (Landino et al., 2021; Sathyan, Ayers, Gao, Milman, et al., 2020). FABP3 and FABP4 are fatty‐acid binding proteins mainly expressed in heart and skeletal muscle and adipocytes, respectively. They are involved in long‐chain fatty acid transportation and lipid metabolism (Furuhashi & Hotamisligil, 2008). Higher FABP4 has been linked to metabolic diseases such as obesity and diabetes and higher FABP3 in the plasma has been linked to fatigue, physical activity intolerance, and muscle atrophy (Dowling et al., 2020; Furuhashi & Hotamisligil, 2008). These two proteins have previously been shown to be associated with frailty index trajectories (Verghese et al., 2021).

We discovered many more proteins cross‐sectionally associated with prefrailty and frailty than with incident frailty. This may partially be the result of greater statistical power with a larger sample size in the cross‐sectional analysis. It may also suggest that many more proteins are dysregulated following (perhaps because of) prefrailty or frailty than are dysregulated preceding frailty. The paradoxical GDF15 findings may exemplify this distinction. GDF15 is a distant member of the transforming growth factor (TGF)‐β superfamily of cytokines. It is released in response to inflammatory stimuli and has an anti‐inflammatory effect (Pence, 2022). It also reduces fat mass by appetite suppression and lipolysis (Fujita et al., 2016). However, higher GDF15 has been consistently shown to be associated with poor muscle health, slower gait speed, and lower physical function (Conte et al., 2020; Semba et al., 2020). Whether these associations are causal remains unknown, but in a recent study, GDF15 failed to show any causal link to 18 aging traits (not including frailty) using Mendelian randomization (Tanaka et al., 2020). In our analysis, GDF15 was associated with incident frailty, providing stronger evidence that GDF15 is involved before the clinical manifestation of frailty. However, this finding may still be driven by the dysregulation of GDF15 among those with elevated inflammatory signaling at baseline, and hence cannot confirm that GDF15 is on the causal pathways of frailty pathogenesis.

Despite our limited ability to delineate the causal roles of the proteins discovered, especially those in the cross‐sectional analysis, the proteins associated with prefrailty and incident frailty may inform biological changes in early stage of frailty and can be targets of interventions to delay or reverse the transition into frailty among prefrail individuals. The smaller overlap of proteins associated with lower odds of prefrailty and frailty at the FDR level (Figure S4) suggests that the protective mechanisms may be more stage‐specific than the pathogenic mechanisms of frailty and the targets for intervention should consider this stage specificity. Given the limitations of current definition of prefrailty (Sezgin et al., 2022), we could not rule out that the proteins associated with prefrailty but not frailty at the FDR level were driven by isolated impairment in frailty component, not necessarily leading to frailty. However, the highly consistent directions of associations with both states among the top proteins (Figure 2e) mitigated such possibility.

Some other notable proteins cross‐sectionally associated with prefrailty and/or frailty in ARIC and replicated in the CHS included growth factors, for example, GHR, IGFBP2, and tumor necrosis factor (TNF) proteins, e.g., TNF receptor superfamily member 1A and 1B (TNFRSF1A, TNFRSF1B) and ligand superfamily member 15 (TNFSF15). GHR and IGFBP2 have both been linked to fat mass, muscle mass, and/or bone density, all important to frailty (Bartke, 2019; van den Beld et al., 2019). TNFRSF1A, TNFRSF1B, and TNFSF15 are all markers of TNF‐mediated inflammation (Jin et al., 2013; Van Epps et al., 2016). The association between inflammation and frailty has been consistently reported in the literature, even though causal evidence is still lacking (Wang et al., 2019). Some proteins were cross‐sectionally associated with prefrailty and/or frailty and the four proteins associated with incident frailty were not replicated in the CHS. This could be due to the differences between the two populations. The participants were recruited from different regions, and the visit used as baseline in CHS was 20 years earlier than the visit used as baseline in ARIC. The regional difference and the change in public health trends over the years may explain the different average BMI and prevalence of chronic conditions (e.g., hypertension and diabetes) and frailty components between the two cohorts (Tables S13 and S14). These factors may influence the levels and biological impact of the discovered proteins. Limited power due to a smaller cross‐sectional sample size and a smaller number of incident frailty cases with shorter follow‐up in CHS (4.0 ± 0.1 years) than in ARIC (6.5 ± 0.7 years) may offer another reason for lack of replication.

Pathway analyses suggest that biological processes related to impaired metabolism, inflammation, and muscle function may be involved in frailty development. Two pathways related to LXR/RXR, molecules that remove cholesterol and other lipids from cells, reduce blood glucose levels (Wente et al., 2007), and inhibit inflammatory signaling (Zelcer & Tontonoz, 2006), were enriched. The LXR/RXR activation pathway was inhibited. The LPS/IL‐1 mediated inhibition of RXR function, a pathway in which pro‐inflammatory cytokines induced by lipopolysaccharide (LPS) reduces RXR levels in the nucleus (Liu et al., 2017), was activated. Axonal guidance signaling is a pathway involved in the process by which axons reach their synaptic targets. It may impact frailty risk via muscle function as suppressed gene expression in muscle tissue of older adults was enriched in this pathway (Turner et al., 2020). The activation of multiple inflammatory pathways suggests the importance of inflammation in frailty, as has been suggested previously (Wang et al., 2019). Unlike previous studies that rely largely on targeted assays of commonly measured inflammatory proteins (e.g., IL‐6, CRP, and TNF‐α), our proteome‐wide approach examined hundreds of immunologically relevant proteins, expanding the evidence for the role of novel inflammatory proteins (e.g., TREM1) and immunologically‐relevant pathways in frailty and pre‐frailty.

Top upstream regulators further suggest that lipid metabolism, angiogenesis, and cell senescence may play a role in frailty development. Though research on SORL1 has focused on its role in Alzheimer's disease via reducing the production of beta‐amyloid (Yin et al., 2015), the overexpression of it in adipose tissue has also been shown to enhance fat deposition (Schmidt et al., 2016). COL18A1 and estrogen receptors regulate many proteins involved in angiogenesis, such as vascular endothelial growth factor (VEGFs), matrix metalloproteinases (MMPs), and metalloproteinase inhibitors (TIMPs) (Losordo & Isner, 2001; Walia et al., 2015). Though COL18A1 inhibits angiogenesis and estrogen promotes angiogenesis, both were predicted to be inhibited in our sample. Angiogenesis is essential for skeletal muscle health because it increases the density of the capillary that supplies blood and oxygen to the muscle (Gu et al., 2006). However, COL18A1 and estrogen receptors measured in plasma may not reflect the abundance in muscle. VEGFA, MMPs, TIMPs, and a few other targets of COL18A1 and estrogen receptors, such as interleukin‐6, C‐X‐C motif chemokine ligand 12, and TNFRSF1A, are SASP factors secreted from senescent cells and are associated with pro‐inflammatory state (Coppé et al., 2010). Therefore, COL18A and estrogen receptors may be involved in frailty development via cell senescence.

Collectively, our findings suggest multiple molecules and biological pathways that should be mechanistically examined as potential targets for interventions to prevent, delay, or reverse frailty. Exercise has been shown to activate frailty‐associated LXR/RXR pathways, inhibit TREM1 pathways (Liberman et al., 2022), increase endostatin levels, and lower VEGF levels (Gu et al., 2004). Caloric restriction could influence frailty through growth hormone suppression (Bartke, 2019), anti‐inflammation, and other mechanisms (Liu et al., 2021).

The strengths of our study include a larger number of community‐dwelling White and Black participants, a broad assessment of the plasma proteome using a highly reliable state‐of‐the‐art proteomic platform, the separation of prefrail and frail states, and the availability of longitudinal follow‐up to capture incident frailty. Our study also has limitations. First, our sample consisted of Black and White participants only. Replication of the results in other races and ethnic groups is necessary to confirm the generalizability of our findings. Second, we had a considerable number of participants who did not return to follow‐up study visits and were excluded from the longitudinal analysis, which may bias our findings. However, many of the covariates we adjusted in the analysis are likely associated with not returning to follow‐up visits and hence may have mitigated some of the bias. Moreover, as participants with worse protein profiles (Figure S6) and frailty were less likely to return to the study visit, we believe the bias was likely conservative. Third, we only examined the transition from the robust or prefrail state to the frail state in the longitudinal analysis but did not explore other transitions such as from robust to prefrail state or from a worse state to a better state. These transitions could be further explored in future studies. Fourth, to control the potential confounding effect of chronic diseases, our fully adjusted results may have inadvertently excluded some mediating effects of the chronic diseases between proteins and frailty. However, the strong correlation of the protein‐frailty associations in models with and without chronic disease adjustments (Figures S1–S3) suggests that adjustment of the diagnosed chronic diseases does not explain away the associations between biological changes related to the diseases and frailty. Lastly, we used a more liberal threshold (nominal *p* < 0.01) when selecting proteins associated with incident frailty for IPA analyses. This may have included some proteins that were associated with incident frailty by chance alone. However, the consistent findings with results of prevalent prefrailty and frailty support the validity of the findings on incident frailty.

In conclusion, we identified a large number of novel proteins associated with prevalent prefrail and frail states and several proteins that predicted the development of incident frailty among older participants in the ARIC study. Pathway and upstream regulator analyses implicated lipid and glucose metabolism, axonal guidance, angiogenesis, inflammation, and cellular senescence as important mechanisms to consider for understanding the pathogenesis of frailty. Many proteins and pathways were dysregulated among prefrail individuals, suggesting they may be the targets for early interventions to prevent frailty. Future studies should investigate whether the proteins discovered in this study have any causal role in frailty development to further elucidate its etiology and examine whether interventions can prevent prefrailty and frailty by regulating these proteins and the pathways in which they are involved.

## AUTHOR CONTRIBUTIONS

F.L., J.S., M.G., J.Co., and K.A.W. conceptualized the study design and analytic choices. F.L., J.Ch., and J.Co. contributed to the data acquisition. F.L. analyzed the ARIC data. T.R.A. performed the replication analysis in the CHS. F.L., J.S., J.W., J.Co., and K.A.W. contributed to the interpretation of the results. All co‐authors provided substantial revisions to the manuscript.

## CONFLICT OF INTEREST STATEMENT

Dr. Josef Coresh is a scientific advisor to Soma Logic. Dr. Bruce M. Psaty serves on the Steering Committee of the Yale Open Data Access Project funded by Johnson & Johnson. Dr. Amil M. Shah receives research support from Novartis through Brigham and Women's Hospital and consultancy fees from Philips Ultrasound and Janssen.

## Supporting information


Figures S1–S6

Tables S1–S18

## Data Availability

Pre‐existing data access policies for each of the parent cohort studies specify that research data requests can be submitted to each steering committee; these will be promptly reviewed for confidentiality or intellectual property restrictions and will not unreasonably be refused. Please refer to the data‐sharing policies of these studies. Individual‐level patient or protein data may further be restricted by consent, confidentiality, or privacy laws/considerations. These policies apply to both clinical and proteomic data.
