# Supplementary material for: Late‐life plasma proteins associated with prevalent and incident frailty: A proteomic analysis
Source: Aging Cell. 2023 Sep 11;22(11):e13975. doi: 10.1111/acel.13975 (PMC10652348; doi:10.1111/acel.13975)
Supplement: Supplementary file 1 — Figures S1–S6 [file ACEL-22-e13975-s002.docx]

Figure S1. Comparison of associations with prevalent prefrailty between Model 1 and Model 2 (left) and between Model 2 and Model 3 (right).


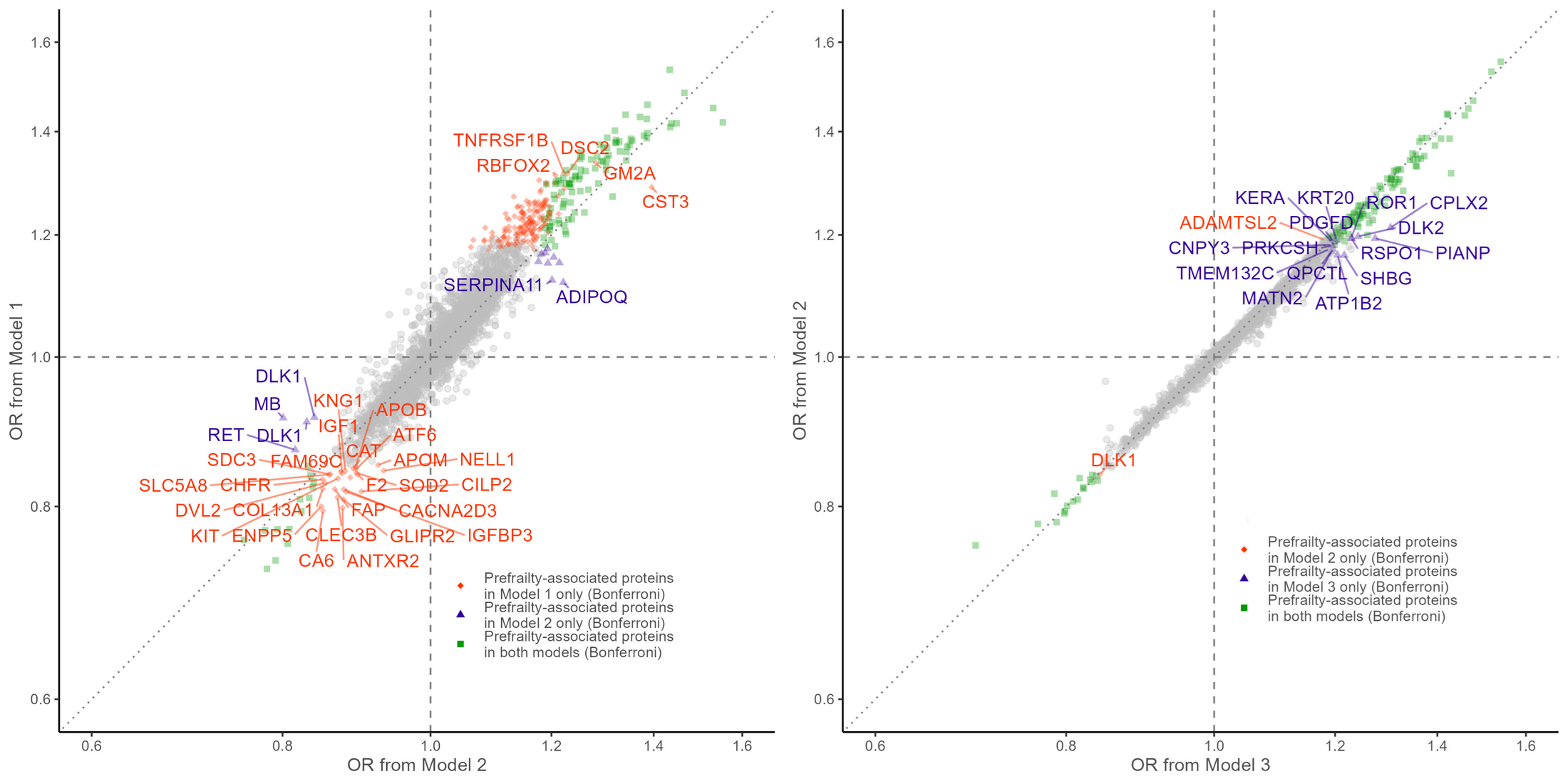


Figure S2. Comparison of associations with prevalent frailty between Model 1 and Model 2 (left) and between Model 2 and Model 3 (right).


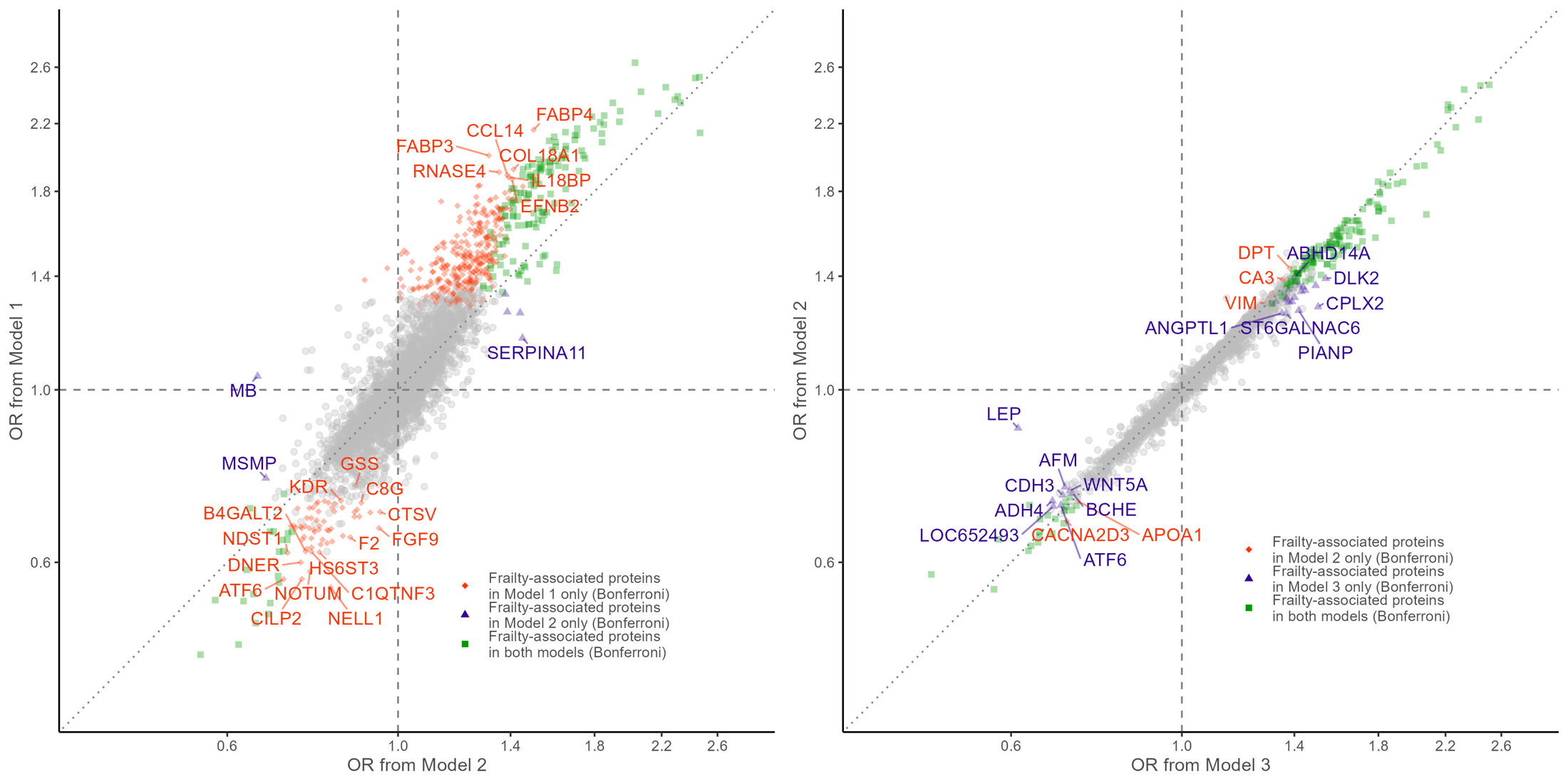


Figure S3. Comparison of associations with incident frailty between Model 1 and Model 2 (left) and between Model 2 and Model 3 (right).


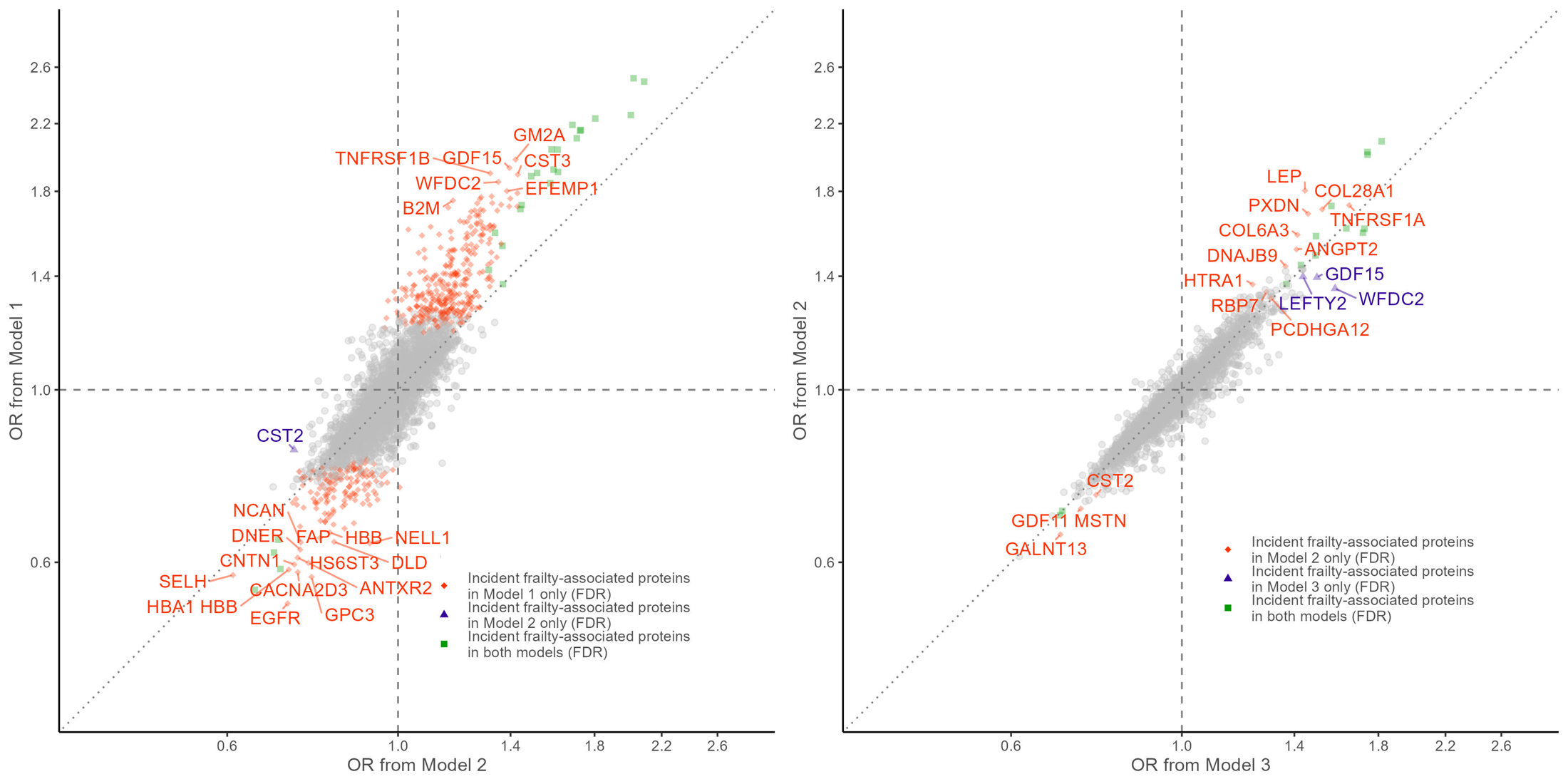


**Figure S4.** Overlap of prefrailty- and frailty-associated proteins at Bonferroni level (A) and FDR level (B) by the directions of association.


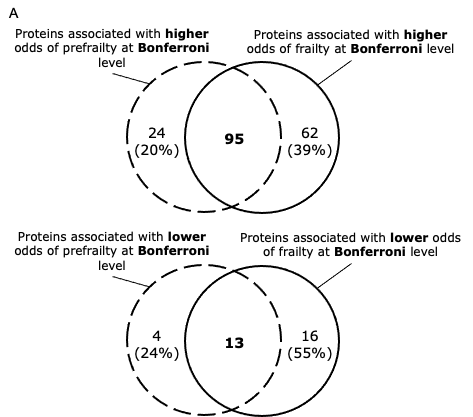


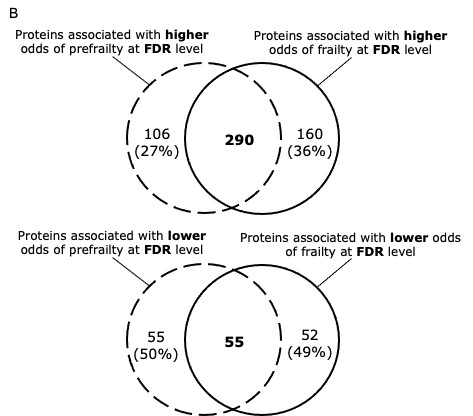


**Figure S5.** The 380 proteins significantly associated with age among the 719 proteins associated with at least one frailty status in our main analyses (i.e., prevalent prefrailty, prevalent frailty, and incident frailty). If a protein was associated with all three states, OR for prevalent frailty was used.


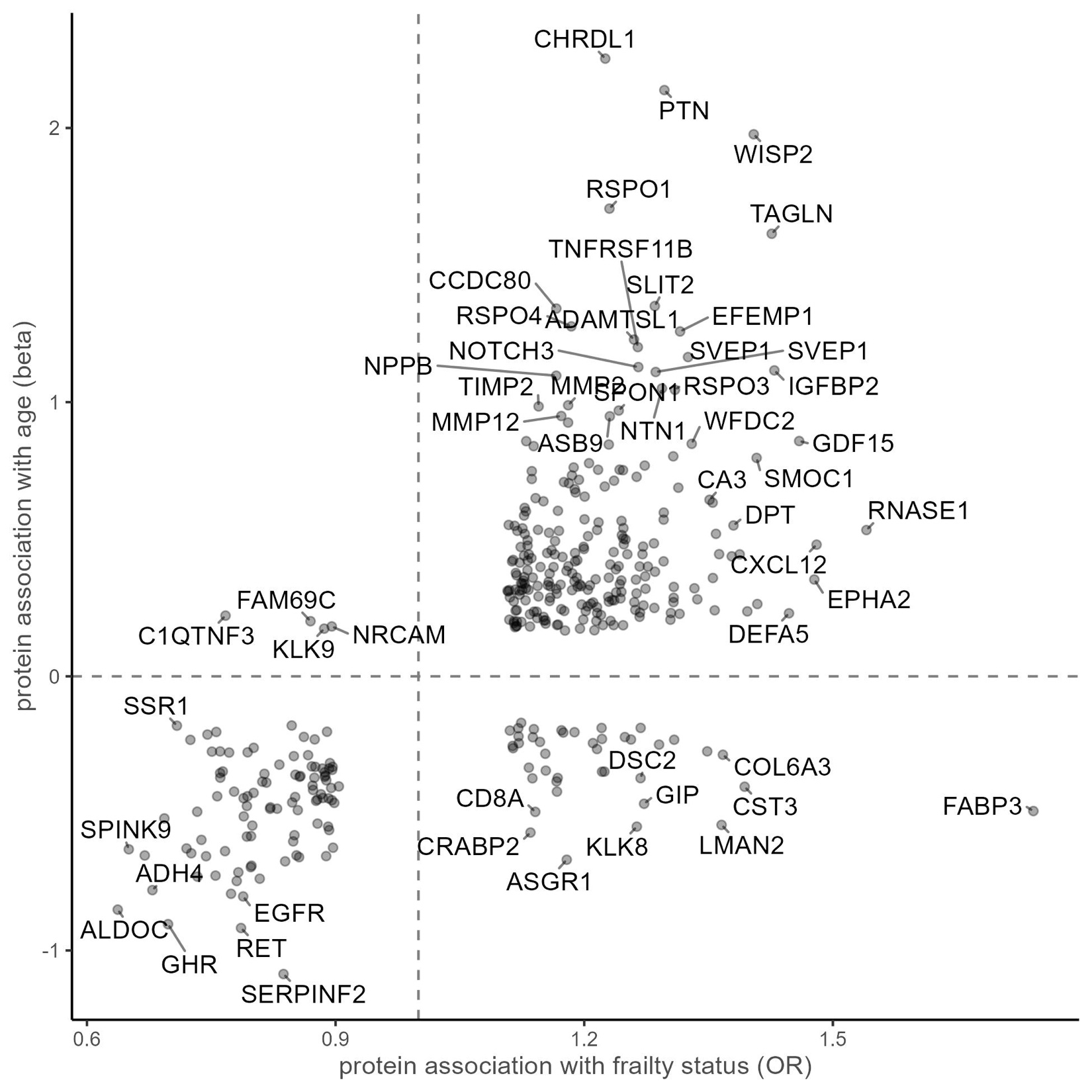


**Figure S6.** The 96 proteins significantly associated with not returning to follow-up visits among the 719 proteins associated with at least one frailty status in our main analyses (i.e., prevalent prefrailty, prevalent frailty, and incident frailty). If a protein was associated with all three states, OR for prevalent frailty was used.

**
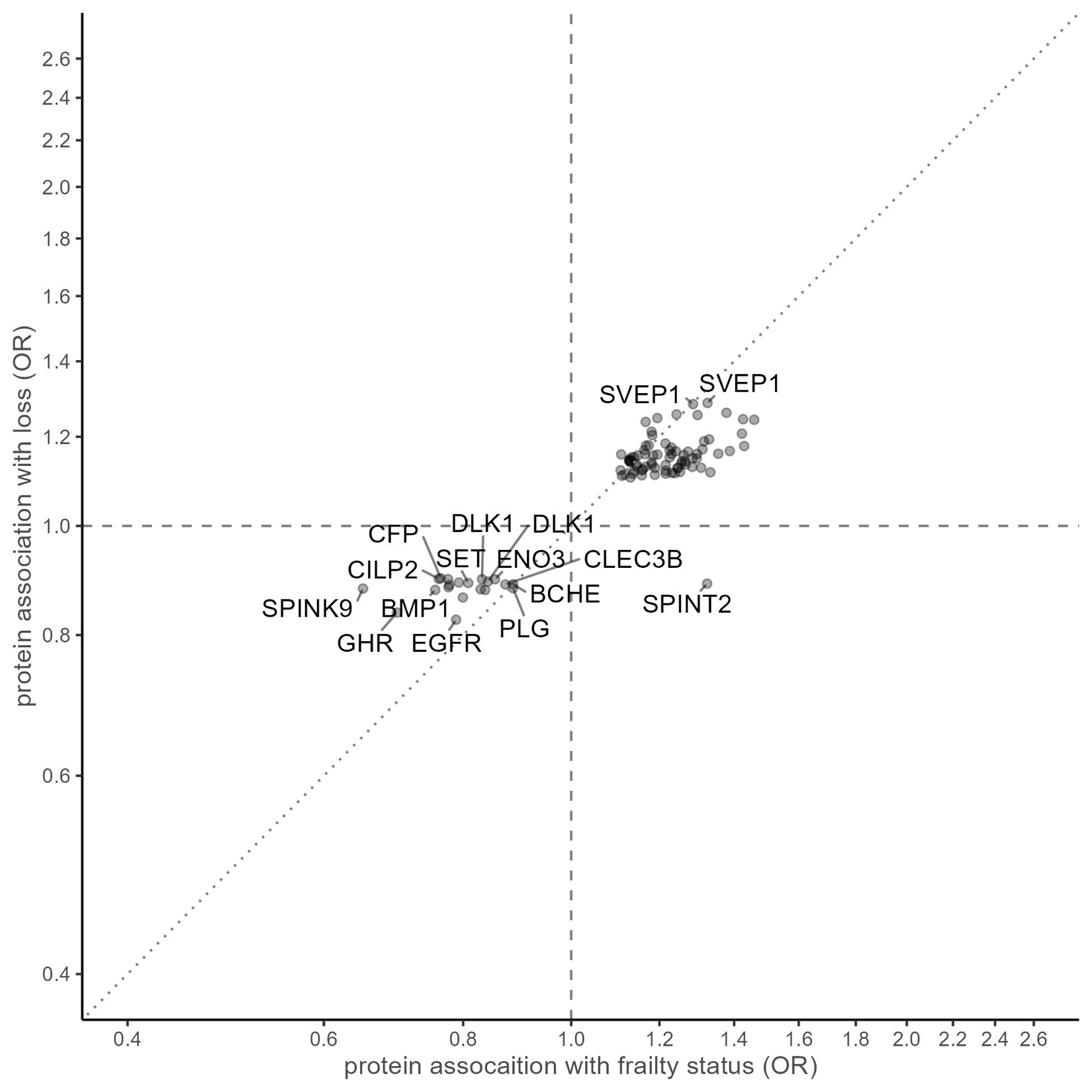
**
